# Parental Rearing Patterns and Interpersonal Skills in Deaf Chinese College Students: The Mediating Role of Theory of Mind

**DOI:** 10.3389/fpsyg.2021.709038

**Published:** 2021-08-20

**Authors:** Yang Wu, Xiping Liu, Shengnan Zhang, Rubo Zhong

**Affiliations:** Faculty of Psychology, Tianjin Normal University, Tianjin, China

**Keywords:** parental rearing patterns, Theory of Mind, interpersonal skills, deaf Chinese college students, gender difference

## Abstract

This study examined the associations between parental rearing patterns and interpersonal skills *via* the mediation of Theory of Mind (ToM) in a sample of 369 deaf Chinese college students. The results showed that negative parental rearing patterns were directly and negatively associated with interpersonal skills, and positive parental rearing patterns were directly and positively associated with interpersonal skills. There were also indirectly associated with interpersonal skills *via* ToM. We also considered whether the mediation of ToM was different for male participants and female participants. The indirect associations between parental rearing patterns and interpersonal skills *via* ToM existed for female participants, but not for male participants. These results indicated that deaf college students’ perceived parental rearing patterns are associated with their interpersonal skills, and parents of deaf children should incorporate ToM in their everyday rearing patterns to improve their children’s interpersonal skills, especially for girls.

## Introduction

Interpersonal skills are an important part of intelligence (Brualdi Timmins, 1996) and were defined as “interpersonal competence” by Klein et al. (2006). Interpersonal skills refer to an individual’s ability to communicate and interact with different objects through the dynamic verbal (and non-verbal) communication means of complex perceptual and cognitive processes. After entering college, students are exposed to various new social environments, and they are provided more opportunities to interact with their peers and strangers. The social network outside the family gradually expands (Bester, 2019). Developing and maintaining dynamic and stable interpersonal relationships become a key task at this stage. Positive interpersonal skills are often associated with several positive outcomes, including wellbeing, a better quality of life, high academic achievements, higher levels of social adjustment performance, and lower mental health problems (Xia et al., 2015; Madison and Coyne, 2017; Zhang and Eggum-Wilkens, 2018). Conversely, poor interpersonal skills are often associated with negative outcomes, such as loneliness, low self-esteem and confidence, and higher levels of mental health problems (Malaquias et al., 2015; Hamilton et al., 2016; Ranta et al., 2016; Owens et al., 2019). Moreover, this negative correlation is more evident in deaf college students. For example, according to Xiong (2015), most interpersonal communication of deaf Chinese students occurs in peer relationships and their interpersonal relationship problems are more severe than those of students with normal hearing. Using a sample of deaf adolescents, Xu (2018) found that deaf adolescents are prone to having conflicts with friends due to the lack of communication skills and their immature concept of friendship, and they are very unlikely to proactively and effectively solve conflicts. With the continuous development of inclusive education, more deaf students live and study with hearing students in colleges. However, due to their deafness, they have difficulties in both general and interpersonal communications. It is very challenging to integrate themselves into society after graduation. Therefore, it is important to study the influencing factors and better understand the mechanisms of interpersonal communication ability in deaf college students.

### Parental Rearing Patterns and Interpersonal Skills

The literature has documented that parental rearing patterns are an important factor affecting interpersonal skills of deaf individuals (Darling and Steinberg, 1993; Huang, 2010; Xiuqin et al., 2010; Llorca et al., 2017). Darling and Steinberg (1993) believe that parental rearing patterns shape the attitude that parents pass on to their children. The emotional climate created by parents’ behavior includes not only behavioral goals but also non-goal-oriented behaviors, such as changes in normal posture, intonation, gesture, and emotions. Parents are children’s first teachers, and their parental rearing patterns will affect children’s personality formation and social interaction (Huang, 2010). For example, adolescents with Internet addiction disorder generally perceive maternal and paternal rearing styles as lacking in emotional warmth, being over-involved, displaying rejection, and being punitive (mothers only). Internet-addicted adolescents also rate higher on obsessiveness, interpersonal sensitivity, depression, and paranoid ideation (Xiuqin et al., 2010). The mother’s permissive style is associated with the increased aggressive behavior and decreased attachment to peers (Llorca et al., 2017).

Parents of deaf children are more likely to adopt inappropriate parental rearing patterns, such as excessive compensation or additional refusal, as a result of their children’s physical disability. They often fail to provide their deaf children appropriate education guidance. Some tend to be more indulgent regarding their children’s education; some are overprotective and participate excessively in their children’s daily lives, negatively impacting the psychological growth of deaf adolescents (Kang, 2018). Gao (2014) found that parents of deaf children pay too much attention to their children’s personal needs and self-care ability instead of their connection with the outside world, thereby hindering the normal social development of deaf children. At the same time, a one-way and forced communication practice makes deaf children most likely to adopt a passive position in interpersonal communication, which is not conducive to their future interpersonal relationships. However, although research has indicated associations between parental rearing patterns and interpersonal skills in deaf adolescents, few studies have examined the relationship in deaf college students.

### The Mediating Role of Theory of Mind

Theory of Mind (ToM) is a term for attributing a psychological state to oneself or others and predicting and explaining human behavior (Premack and Woodruff, 1978), the ability to understand the inner world of others. Social constructivism holds that children construct their understanding of others by participating in social interaction mediated by language (Fernyhough, 2008). This social interaction includes the interaction between parents and children. It is possible that ToM mediates the relationship between parental rearing patterns and interpersonal skills. First, some studies have shown that ToM is a key factor in the development of interpersonal skills and in the formation and maintenance of social relations (Devine and Hughes, 2016; Lecciso et al., 2016; Petrocchi et al., 2021). For example, in kindergarteners, higher ToM was related to stronger social skills (Razza and Blair, 2009). Poor ToM may lead to being a victim or a bully in early adolescence (Happé et al., 2013). Second, some studies have shown that proper parenting styles include emotional involvement and equal dialogue with children; however, inappropriate parenting styles (such as high involvement or rejection) make it difficult to have such a dialogue between parents and children (Fuentes et al., 2015; Lorence et al., 2020). Verbal dialogue is an essential part of parenting (Keller et al., 2008).

Studies have also shown that dialogue between family members is helpful for adolescents to think in transposition (Weber and Carter, 1998). Children who disclose personal information to family members receive high scores in the ToM task (Rotenberg, 2010). When family conversations involve states of mind, there will be discussion on the differences in the thoughts of oneself and others. Children are aware of thoughts, memories, and beliefs, and all of these affect the development of ToM (Dunn et al., 1991). However, this kind of family dialogue raises questions as to whether there is a common language in the family and in the rearing patterns of parents of deaf children. First, deaf children who grow up in a hearing environment may not have a common language at home; they may not be able to use sign language to communicate. This would result in limited opportunities for deaf children to participate in various dialogues, which, for example, include different views and use of mental state terms (such as know, believe, and think; Moeller and Schick, 2006), and a series of syntactic structures (De Villiers and De Villiers, 2014). Although many deaf children’s parents also learn sign language, they may not be fluent in it. Thus, the early language environment of deaf children may not be as good as that of hearing children (Moeller and Schick, 2006). Compared with deaf children growing up in deaf families, deaf children growing up in hearing families have less conversation at home (e.g., Peterson and Slaughter, 2006). Parents’ conversation with deaf children lacks high-level facilitating discourse (such as the use of psychological terms) and is more indicative than their conversation with hearing children (Ambrose et al., 2015). This situation often results in deaf children being unable to talk freely to their parents about their observations, feelings, and other psychological states. Their ToM can be as retarded as that of autistic children. Second, parents may not have much patience to build a warm upbringing environment in which their deaf children could discuss their psychological state, and that of others. It may be difficult for parents who often refuse and deny their children and are indifferent to their children’s feelings to create a family environment where they could have a psychological dialogue. This study aimed to examine whether ToM would mediate the relationship between parental rearing patterns and interpersonal skills among deaf Chinese college students. Such a model would make a good contribution to the literature.

### Gender Difference

Studies have indicated gender differences in ToM at children’s different stages of development. Preschool girls have shown a slight advantage over boys in emotional understanding and false belief tasks (Banerjee, 1997; Charman et al., 2002; Walker, 2005). Xu and Ge (2010) used low-level and high-level ToM stories to test whether there were gender differences in college students’ ToM levels. They found that there were significant gender differences in low-level tasks (but not in high-level tasks); that is, girls’ understanding of other people’s beliefs and white lie stories was better than that of boys. Wu (2014) tested ToM levels of adults at different age stages and found that females’ scores of ToM were higher than those of males in all three age groups (youth, middle age, and old age) and that there was gender difference in young people’s ToM (but not in the other two age groups). Additionally, this gender difference was confirmed in neuroimaging. Researchers (e.g., Derntl et al., 2010; Gao et al., 2019) divided ToM into cognitive dimension (cognitive ToM/cToM) and affective dimension (affective ToM/aToM) and tested gender differences in brain nerve responses in these two dimensions in adolescents. According to Gao et al. (2019), when completing the cognitive ToM task, the activity of left temporoparietal junction in male adolescents was significantly higher than that of female adolescents. Derntl et al. (2010) found that when inferring emotional responses from graphic scenes, men were more likely than women to activate the temporoparietal junction. However, when inferring emotions, women were more likely than men to activate the amygdala, inferior frontal gyrus, and superior temporal sulcus. This study considered whether there would be gender differences, if our proposed mediation model *via* ToM existed.

### The Present Study

The present study was designed to examine the associations between parental rearing patterns and interpersonal skills within a sample of Chinese deaf college students. We hypothesized that negative parental rearing patterns will be negatively associated with interpersonal skills, whereas positive parental rearing patterns will be positively associated with interpersonal skills. We also considered whether the relationship between parental rearing patterns and interpersonal skills would be mediated *via* ToM. We expected that deaf college students who reported lower perceived negative parental rearing patterns would report higher ToM, which in turn would be positively associated with interpersonal skills. Higher positive parental rearing patterns were expected to be associated with increased interpersonal skills due to its association with ToM. We also considered whether the relationship between parental rearing patterns and interpersonal skills *via* ToM was different for male participants and female participants but offered no hypothesis.

## Materials and Methods

### Participants and Recruitment

The sample included 369 deaf college students (*M* = 19.94 years old, *SD* = 1.52; 50.1% female) at a special education college in the northern China. During the Spring 2020 semester, 380 hearing-impaired students were invited to participate in a research study of student behaviors and beliefs. To be eligible, students had to be 17 years or older with hearing loss. They had to have passed the college entrance test and have a proficient level of written and spoken Chinese or Chinese sign language. All students had passed the admission mental health examination and had not been clinically diagnosed with any mental disorder. Eleven students did not attend the study.

### Procedure and Measures

Student assent and written informed consent from parents were obtained. Participation in the study was voluntary, and no economic remuneration was provided. After providing the informed consent, students proceeded to fill the paper-and-pencil survey questionnaire during the regular class hours. Participants were randomly assigned to either classrooms. Questionnaires were administered in each of these classrooms by two trained researchers and one interpreter proficient in Chinese sign language. We believed that this process would reduce the pressure of sign language translation and improve the testing quality. When the deaf college participants had difficulties with the written language, they could seek help from the sign language interpreter. The survey questionnaire was completed in 60 min. All procedures were approved by the University Research Ethics Board (Institutional Review Board).

**Parental rearing patterns** were measured using the adapted version of the Short-Egna Minnen av Barndoms Uppfostran (S-EMBU; Arrindell et al., 1999; Jiang et al., 2010). The S-EMBU includes 21 items and assesses students’ perceptions of their mother’s and father’s behavior toward them. These included six items on rejection (e.g., “My father/mother punishes me for even the smallest mistakes.”), eight items on overprotection (e.g., “I feel my parents interfere in everything I do.”), and seven items on emotional warmth (e.g., “Father/mother praises me.”), rated on 4-point scales, from never (1) to always (4). We took the average of the rejection and overprotection items to compute a negative parental rearing patterns variable (*α* = 0.83), and the emotional warmth items were averaged to create a positive parental rearing patterns variable (*α* = 0.86) for each participant.

**Interpersonal skills** were measured using the 40-item Interpersonal Competence Questionnaire developed by psychologists from the University of California (Wei, 2005). The questionnaire assesses college students’ proactive communication (eight items; e.g., “Start a conversation with a stranger you’d like to meet.”), appropriate refusal (eight items; e.g., “Reject unreasonable demands from people close to you.”), self-disclosure (eight items; e.g., “Tell your loved ones something about yourself that you are ashamed of.”), conflict management (eight items; e.g., “Able to open up feelings of jealousy or resentment during an argument with someone close to you.”), and emotional support (eight items; e.g., “To help close people think and experience their major life decisions.”), rated on a 5-point scale, from 1 (can’t do, feel extremely uneasy and not sure, and try to escape) to 5 (very good at doing this, feel very relaxed, and can handle very well). We took the sum of all these 40 items to create an interpersonal skills variable for each participant (*α* = 0.92).

**Theory of Mind** was measured using the adapted version of the advanced Theory-of-Mind tasks (AToM; Osterhaus et al., 2016). The AToM assesses participants’ social reasoning (nine items; e.g., “Why does Mrs. Smith say that?”), fuzzy reasoning (three items; e.g., “What did Maomao see in the picture?”), and the understanding of deviant behavior from social norms (three items; e.g., “Did Johnny remember the birthday party was a surprise?”). The scale consists of a series of ToM stories and pictures, and following each of these, one or more questions were provided. There were 15 questions in total, and the responses were Yes (1) or No (0). We summed all these 15 responses to produce a ToM score for each participant (*α* = 0.70).

### Analytic Plan

**Figure 1:**
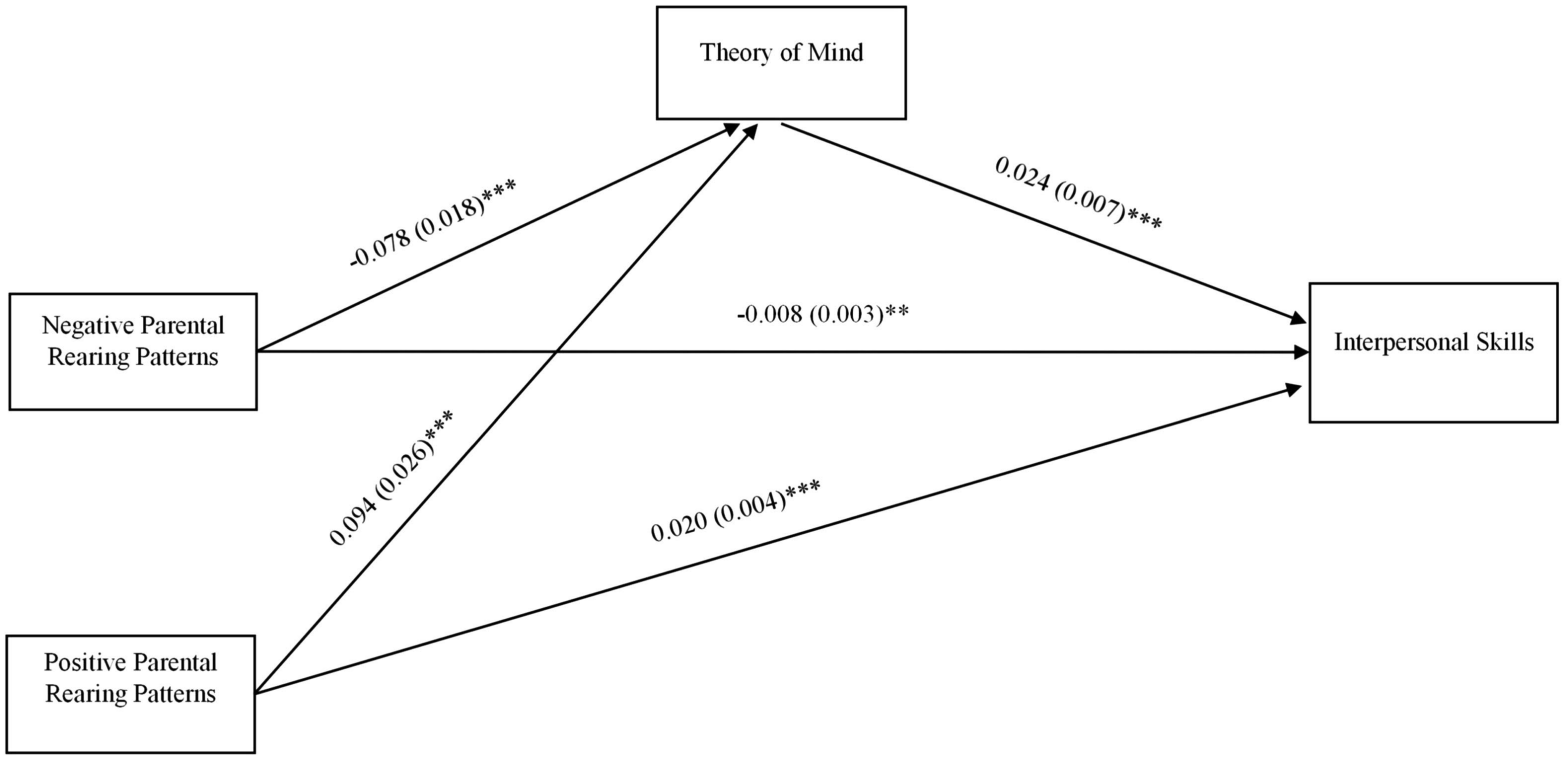
Indirect effects of parental rearing patterns on interpersonal skills via Theory of Mind (ToM). Path coefficients (b and standard error, SE) are unstandardized. Model controlled for potential effects of gender and age on ToM and interpersonal skills. ^***^p < 0.001; ^**^p < 0.01; ^*^p < 0.05.

We hypothesized that negative parental rearing patterns would be negatively associated with deaf college students’ interpersonal skills, whereas positive parental rearing patterns would be positively associated with their interpersonal skills. We also hypothesized that the relationship between parental rearing patterns and interpersonal skills would be mediated *via* ToM. We tested the direct and indirect effects of parental rearing patterns on interpersonal skills in Mplus Version 8.6 (Muthén et al., 2016; Muthén and Muthén, 2017). We used maximum likelihood estimation with robust standard errors. The statistical significance of the indirect effect was tested using 50,000 bootstrap draws to estimate precisely the 95% confidence intervals determined from the lower and upper 2.5 percentiles (MacKinnon et al., 2004; Hayes, 2013). Participants’ gender (0 = male; 1 = female) and age were included as covariates, allowing us to control for their influence on the outcome (i.e., interpersonal skills) and on the mediator (i.e., ToM) in the model (see Figure 1). We also considered whether the paths from parental rearing patterns to interpersonal skills *via* ToM were different for male participants and female participants. Thus, we conducted multiple group analysis (Muthén and Muthén, 2017).

## Results

### Descriptive Data

The descriptives and bivariate correlations among primary variables are displayed in Table 1. Independent samples t-test did not indicate gender differences in the primary variables, that is, interpersonal skills, perceived parental rearing patterns, ToM, or age.

**Table 1:** Descriptives.

S. No.	Variable	Min	Max	Mean	SD	1	2	3	4	5	6
1.	Interpersonal skills	1.73	4.70	3.22	0.54	–					
2.	Negative parenting style	32.00	80.00	54.52	9.64	−0.22^**^	–				
3.	Positive parenting style	14.00	56.00	38.77	7.36	0.33^**^	−0.14^**^	–			
4.	Theory of Mind	4.00	24.00	15.63	3.78	0.27^**^	−0.23^**^	0.23^**^	–		
5.	Age	17.00	23.00	19.94	1.52	0.11^*^	−0.03	0.12^*^	0.23^*^	–	
6.	Gender	0.00	1.00	0.50	0.50	0.00	0.04	−0.02	−0.04	−0.03	–

** *Correlation is significant at the 0.01 level (two-tailed);*

* *Correlation is significant at the 0.05 level (two-tailed)*.

### Are the Effects of Parental Rearing Patterns on Interpersonal Skills Mediated *via* ToM?

We tested the direct and indirect effects of perceived negative parental rearing patterns and positive parental rearing patterns on interpersonal skills. As shown in Figure 1, negative parental rearing patterns were negatively associated with ToM, which in turn was associated with higher interpersonal skills. The indirect effect of negative parental rearing patterns *via* ToM was significant, *b* = −0.002 (0.001), *p* = 0.007, and 95% bootstrap confidence intervals did not include the value of 0 (−0.003, 0.000), *b* = −0.002 (0.001), *p* = 0.009. Negative parental rearing patterns were also directly and negatively associated with interpersonal skills, *b* = −0.008 (0.003), *p* = 0.002. The total estimated effect from negative parental rearing patterns to interpersonal skills was *b* = −0.010 (0.002), *p* < 0.001. Positive parental rearing patterns were positively associated with ToM, which in turn was associated with higher interpersonal skills. The path *via* ToM was significant, *b* = 0.002 (0.001), *p* = 0.011, and 95% bootstrap confidence intervals did not include the value of 0 (0.000, 0.004), *b* = 0.002 (0.001), *p* = 0.013. Positive parental rearing patterns were also directly and positively associated with interpersonal skills, *b* = 0.020 (0.004), *p* < 0.001. The total estimated effect from positive parental rearing patterns to interpersonal skills was *b* = 0.022 (0.004), *p* < 0.001. The model controlled for gender and age on the outcome (i.e., interpersonal skills) and the mediator (i.e., ToM). The effect of gender was not significant on ToM (*p* = 0.684) or on interpersonal skills (*p* = 0.696). Age had a significant effect on ToM, *b* = 0.508 (0.129), *p* < 0.001, but not on interpersonal skills (*p* = 0.509). The model fit indices were good: *χ*^2^(4) = 5.721, *p* = 0.221; CFI/TLI = 0.986/0.968; RMSEA = 0.034; SRMR = 0.031.

### Gender Difference in the Mediation Role of ToM

**Figure 2:**
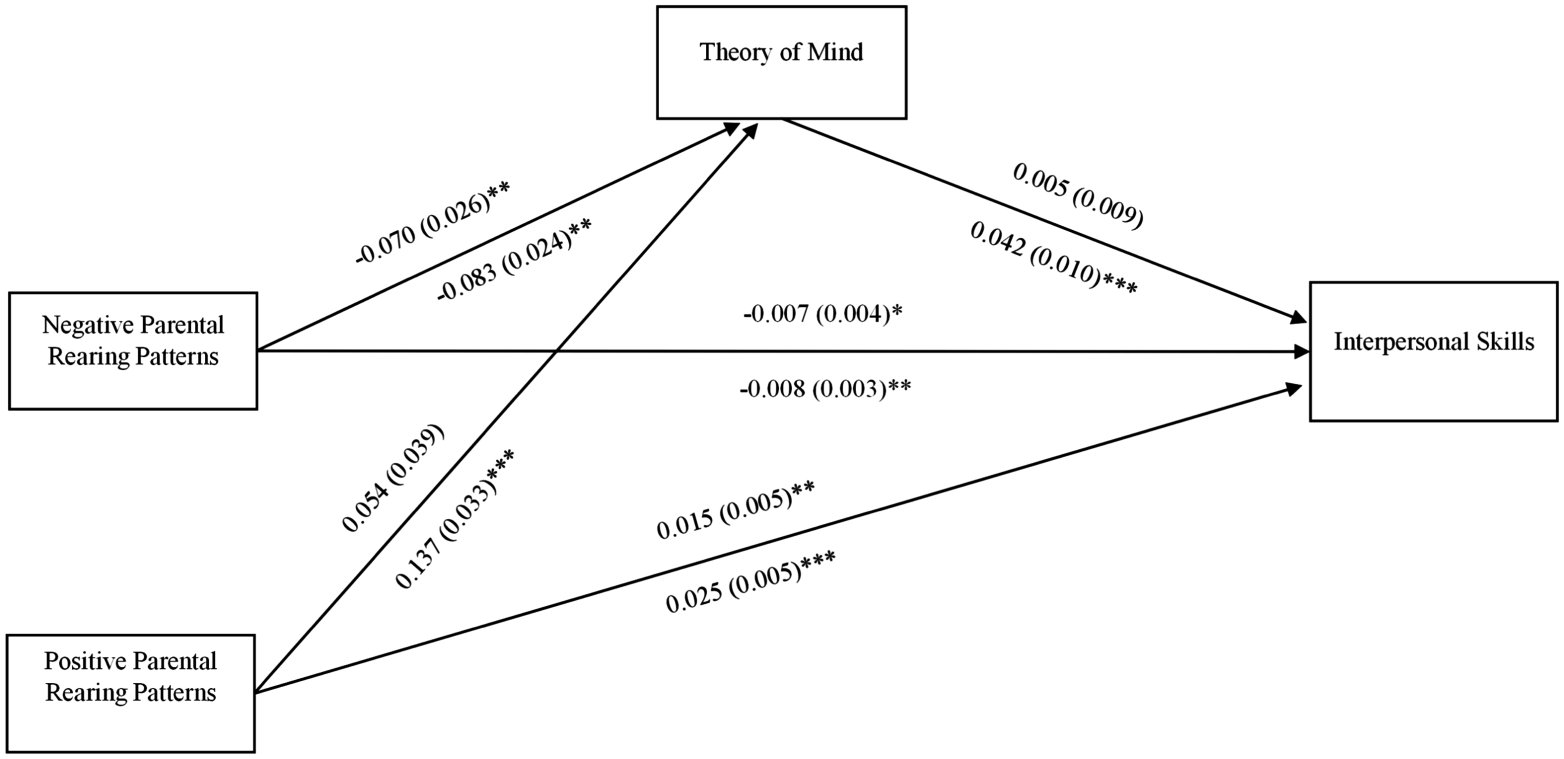
Indirect effects of parental rearing patterns on interpersonal skills *via* ToM, by gender. Path coefficients (b and standard error, SE) are unstandardized. Path coefficients above the arrows were for male participants, and those below the arrows were for female participants. Model controlled for potential effect of age on ToM and interpersonal skills. ^***^p < 0.001; ^**^p < 0.01; ^*^p < 0.05.

We conducted multiple group analysis to examine whether the paths *via* ToM were different for male participants and female participants. Results showed good model fit: *χ*^2^(4) = 5.121, *p* = 0.275; CFI/TLI = 0.993/0.975; RMSEA = 0.039; SRMR = 0.035. As shown in Figure 2, for female participants, ToM mediated the relationship between negative parental rearing patterns and interpersonal skills, *b* = −0.004 (0.001), *p* = 0.008 (95% bootstrap confidence intervals were −0.006, −0.001) and also the relationship positive parental rearing patterns and interpersonal skills, *b* = 0.006 (0.002), *p* = 0.001 (95% bootstrap confidence intervals were 0.002, 0.010). ToM did *not* mediate the path from negative parental rearing patterns to interpersonal skills or from positive parental rearing patterns to interpersonal skills for male participants.

## Discussion

This study examined the relationship between parental rearing patterns and interpersonal skills and its mechanism *via* ToM in a sample of deaf Chinese college students. Firstly, parenting styles were significantly associated with ToM, which in turn was associated with higher interpersonal skills; Secondly, ToM played a mediating role in the relationship between parental rearing patterns and interpersonal communication ability. However, multiple grouping modeling indicated that this mediation role of ToM was observed only for female participants (but not for male participants).

### Relationship Between Parental Rearing Patterns and Interpersonal Communication Ability in Deaf College Students

This study found that parental rearing patterns were significantly associated with interpersonal ability of deaf Chinese college students. Positive parental rearing patterns were positively associated with interpersonal skills, while negative parental rearing patterns were negatively associated with interpersonal skills. The results are consistent with previous studies (Liu et al., 2018). Social learning theory holds that children interact directly with parents by observing their parents’ interaction patterns or by learning from parents under their guidance (Crittenden, 1984). The learning includes attitudes, values, personality, behavior, and social emotions (Dohmen et al., 2012; Schofield et al., 2012; Barni et al., 2013). Parents’ rearing patterns are an interactive way for children to learn from their parents. Through this interaction, parents convey their interpersonal relationship model to their children. Thus, interpersonal and intergenerational transmission occurs.

Some studies have shown that the parent–child relationship and especially the fathers’ rearing pattern explain the advantages of children’s interpersonal relationships (Conger et al., 2010). Parents with emotional warmth will show an interest in their children’s activities and opinions. They will talk about their feelings and emotions with them, provide comfort, moral guidance, and advice, participate in social activities with them, listen to them carefully, and help them solve problems. This warm upbringing approach is related to positive mental health, social ability, self-esteem, academic achievement, and healthy peer relationships (Boudreault-Bouchard et al., 2013; Grolnick et al., 2015). The more childhood memories of parental rejection individuals have, the more likely they are to experience psychological disorders (Rohner and Khaleque, 2010), and these negative memories will compromise the development of their interpersonal ability. Such conclusions have also been demonstrated in cross-cultural studies (Ali et al., 2015).

### Mediating Role of ToM and the Gender Difference

This study found that ToM plays a mediating role in the relationship between parental rearing patterns and interpersonal communication ability in deaf college students. This suggests that parents’ positive parental rearing patterns are conducive to improving the level of ToM of deaf college students, which will make it easier for them to transpose thinking and to understand individual psychological differences by observing other people’s external behavior. This will help them to improve their interpersonal skills and to conduct interpersonal communication calmly and appropriately. However, deaf college students’ perceived negative parental rearing patterns will lead to the lagging development of their ToM, making it difficult to integrate themselves into normal interpersonal relationships.

In families with deaf children, due to the physical disability of the children, parents may be prone to overintervention and overprotection in their parenting because they may experience guilt about their children. In addition, deaf children growing up in hearing families may have difficulty in effective communication to promote psychological growth due to the lack of a common language with their parents. Studies have shown that communication with parents is an important predictor of group identification and socialization standard for deaf persons (Calderon, 2000). The development of ToM is influenced by a number of external environmental factors, among which the family environment is particularly important. Devine and Hughes (2016) concluded in a meta-analysis that parents’ psychological conversation and their way of thinking are significantly correlated with their children’s ToM level. Even after controlling for the language factors, these correlations still exist. Social constructivist theorists believe that increasing the discussion of mental states provides children with more opportunities to understand other people’s thoughts, thereby testing and revising their ToM (Jenkins et al., 2003; Wellman, 2017). The improvement and development of ToM skills can support the ability to sympathize with others (Peterson, 2016), help individuals to have more friends (Peterson and Siegal, 2002; Fink et al., 2015), and even affect peer popularity (Peterson, 2016).

This study also found that ToM mediated the relationship between negative/positive parental rearing patterns and interpersonal skills only for female deaf college students. There are two possible reasons for this disparity. First, girls’ understanding of psychological theory has been ahead of boys since childhood (Walker, 2005; Wu, 2014). Behavioral studies show that women perform better than men in social sensitivity, empathy, and emotional intelligence tasks (McClure, 2000; Baron-Cohen and Wheelwright, 2004; Brackett and Salovey, 2006). These abilities are closely related to the level of ToM. Gender difference in ToM is also reflected in brain activity. Neuroimaging studies have shown that compared with men of the same age, women use additional brain regions in the ToM task for emotion and self-reference thinking (Christov-Moore et al., 2014). Frank et al. (2015) found that in the process of false belief reasoning, women are more likely than men to activate the medial prefrontal cortex. Second, Chinese social culture requires men to appear stronger and more independent. Therefore, parents are more inclined to adopt a free-range parental rearing patterns for boys. As a result, parental rearing patterns may have no significant impact on boys’ interpersonal skills. When girls are young, they have more advantages in emotion recognition and expression than boys. Gender differences in the level of ToM may further promote the interaction between parents and girls, and girls may gain more attention from parents, resulting in significant gender differences in the mediating role of ToM between parental rearing patterns and interpersonal skills.

### Limitations and Prospects

There are some limitations in this study. First, the relationship model in this study was based on a cross-sectional design. Future studies should collect multiple points of data and combine both vertical and horizontal research results to replicate and better understand the proposed relationship model which we examined in a sample of deaf Chinese college students. Second, this study showed gender differences in the mediating effect of ToM; that is, ToM mediated the relationship between parental rearing patters and interpersonal skills only for female participants. However, future research should replicate it and we also encourage these findings should be replicated in adolescents. Third, the data in this study were participants self-reported. Future research could use a variety of data collection methods to obtain more accurate results, for example, including the perceptions of parents themselves. Finally, the data were collected from deaf students at only one college. The findings should be interpreted with caution.

### Educational Enlightenment

First, schools should increase their interactions with the parents of the students with hearing loss and better understand these students’ parental rearing patterns and their influences on their children’s everyday practice. Parents should be respectfully educated about inappropriate parental rearing patterns using appropriate, evidence-based programs. Second, deaf college students should be trained with a special attention paid to the improvement of their ToM, which could be implemented in a targeted and phased way through daily management and group counseling. For example, for those deaf students whose ToM’ level is lagging, targeted training should be available to help them to integrate better into society. Third, parents of deaf children should be educated to avoid adopting overprotective parental rearing patterns with scientific research evidence. In rearing deaf children, parents should be encouraged to learn sign language as much as possible, and their children should wear a hearing-aid/cochlear implantation (CI) as early as possible, thereby reducing the communication barriers between parents and children. Furthermore, parents should be encouraged to pay attention to the development of children’s ToM and help children to improve the level of ToM through effective communication. Social support should be available for these parents and families. Finally, although this study did not observe a significant mediating role of ToM for male students, and for female and male deaf college students, different management and guidance methods may be adopted according to their individual characteristics, we believe that warm teacher–student relationships and peer relationships can help students to integrate into groups and can improve their interpersonal skills.

## Data Availability Statement

The raw data supporting the conclusions of this article will be made available by the authors, without undue reservation.

## Ethics Statement

All procedures were approved by the University Research Ethics Board (Institutional Review Board). Student assent and written informed consent from parents were obtained. Written informed consent was obtained from the individuals for the publication of any potentially identifiable images or data included in this article.

## Author Contributions

YW analyzed the data and wrote the materials and methods, results, and discussion sections. XL and SZ contributed to the conception and design of the study. RZ is responsible for the drafting the work and revising it critically for important intellectual content. All authors contributed to the article and approved the submitted version.

## Conflict of Interest

The authors declare that the research was conducted in the absence of any commercial or financial relationships that could be construed as a potential conflict of interest.

## Publisher’s Note

All claims expressed in this article are solely those of the authors and do not necessarily represent those of their affiliated organizations, or those of the publisher, the editors and the reviewers. Any product that may be evaluated in this article, or claim that may be made by its manufacturer, is not guaranteed or endorsed by the publisher.
